# Hyaluronate Acid-Dependent Protection and Enhanced Corneal Wound Healing against Oxidative Damage in Corneal Epithelial Cells

**DOI:** 10.1155/2016/6538051

**Published:** 2016-04-14

**Authors:** Jing Zhong, Yuqing Deng, Bishan Tian, Bowen Wang, Yifang Sun, Haixiang Huang, Ling Chen, Shiqi Ling, Jin Yuan

**Affiliations:** ^1^State Key Laboratory of Ophthalmology, Zhongshan Ophthalmic Centre, Sun Yat-sen University, Guangzhou 510000, China; ^2^Department of Ophthalmology, The Third Affiliated Hospital, Sun Yat-sen University, Guangzhou 510000, China

## Abstract

*Purpose*. To evaluate the effects and mechanism of exogenous hyaluronate (HA) in promoting corneal wound healing.* Methods*. Human corneal epithelial cells (HCECs) were incubated with different concentrations of HA to evaluate their efficiency in promoting cell migration and their modulation of repair factors. After inducing hyperosmolar conditions, the cell morphologies, cell apoptosis, and expression levels of TNF-*α* and MMP-9 were detected to assess the protective role of HA. Corneal epithelium-injured rat models were established to test the therapeutic effects of 0.3% HA. Then, the wound healing rates, the RNA expression levels of inflammatory cytokines, and repair factors were examined.* Results*. HCECs in the 0.03% and 0.3% HA groups showed fewer morphological alterations and lower rates of cell apoptosis following preincubation with HA under hyperosmolar conditions, as well as the expression levels of MMP-9 and TNF-*α*. In the rat model, the areas of fluorescein staining in the corneas of 0.3% HA group were significantly smaller than the control group. The expression levels of IL-1*β* and MMP-9 were decreased, while CD44 and FN were increased in the 0.3% HA group.* Conclusion*. HA enhanced corneal epithelial cell wound healing by promoting cell migration, upregulating repair responses, and suppressing inflammatory responses.

## 1. Introduction

A healthy corneal epithelium is essential to protect the eye against infection and structural damage [1]. The conditions/factors that most commonly lead to epithelial defects include epithelial stem cell deficiency, inflammatory diseases, neurotrophic diseases, and mechanical factors [2–4]. Several clinical treatments are used for epithelial defects, including lubrication, punctual plugs, bandage contact lenses, and tarsorrhaphy [5, 6]. Specifically, artificial tears represent one of the most important interventions for lubricating the ocular surface, supplementing insufficient tears, diluting inflammatory cytokines, and reducing the tear osmotic pressure, which has the potential to induce cell apoptosis [7, 8].

Hyaluronate is an excellent representative of artificial tears, and it is used to alleviate ocular discomfort, prolong tear stability, and promote corneal epithelial repair [9]. It is a glycosaminoglycan found in various connective tissues, such as epithelial and neural tissues, and it interacts with water to dilate the extracellular matrix and acts as a lubricant to assist in cell migration [10]. According to the published reports, its concentration increases during the process of wound repair, and it is used as an exogenous intervention to promote this process [11]. The role of HA as a key component of the extracellular matrix structure has been recognized for many decades [12], while its actions on cells involved in corneal epithelial repair have been determined in part only in the last few years [13].

Hyaluronate, as well as its degradation products that are generated during corneal epithelial repair, is capable of activating specific intracellular responses, of which epithelial proliferation, cell apoptosis, inflammatory responses, and neovascularization have been exclusively examined by* in vitro* studies [14, 15]. The molecular mechanisms leading to cell activation have been substantially clarified, and it is now widely accepted that the cellular actions of hyaluronate are mediated by specific surface receptors, including CD44, Fibronectin, RHAMM, and Toll-like receptors [16, 17]. In 1996, Miyazaki et al. reported that hyaluronate binds to the CD44-like molecule associated with FN and enhances the growth of corneal epithelial cells [12]. The latest reports published in 2015 have demonstrated that hyaluronate stimulates the reepithelialization of corneal wounds* in vitro* [4]. However, the mechanism and effects of exogenous hyaluronate remain to be further elucidated, which will allow for the optimization of its clinical use.

Our study aimed to examine the characteristics of hyaluronate and the process of corneal epithelial cell repair to elucidate the mechanism by which exogenous hyaluronate promotes the healing of injured corneal epithelial cells and to determine the optimal concentration of hyaluronate for clinical use. Moreover, we generated a corneal epithelium-injured animal model to analyse the therapeutic effects and mechanism of hyaluronate and to provide experimental and theoretic evidence for managing corneal epithelial defects clinically.

## 2. Materials and Methods

### 2.1. Cell Culture

The human corneal epithelial cell (HCEC) line was a gift from Professor ZhiChong Wang (Zhongshan Ophthalmic Centre, Guangzhou, China). The cell line was maintained in Dulbecco's-modified Eagle's medium (Gibco BRL) supplemented with 15% foetal bovine serum (Gibco BRL), 10 ng/mL human EGF (Gibco BRL), 5 mg/mL insulin, 5 mg/mL human transferase (Sigma), 0.4 mg/mL hydrocortisone (Gibco BRL), 0.1 mm 2-mercaptoethanol (Gibco BRL), 2 mM L-glutamine, 100 U/mL penicillin, and 100 mg/mL streptomycin (HyClone; corneal epithelial cell culture medium).

### 2.2. Scratch Wound Healing Assay

HCECs were trypsinized and seeded at a density of 10,000 cells per well into 24-well plates that contained a culture insert. Then, scratch wounds and premarked lines were created after a 24 h incubation period. After the removal of the debris resulting from the linear scratching, HCEC monolayers were divided into the following three groups: a control group, which was incubated with serum-free medium for 48 h; a 0.03 HA group, which was incubated with serum-free medium containing hyaluronate at a final concentration of 0.03% for 48 h; and a 0.3 HA group, which was incubated with serum-free medium containing hyaluronate at a final concentration of 0.3% for 48 h. Cell proliferation was recorded using an inverted microscope, and images were captured to visualize the interactions between the scratched wound areas and premarked lines. Then, the protein levels of the repair factors FN and CD44 were measured by ELISA.

### 2.3. Cell Challenge Conditions

HCECs were trypsinized and seeded at a density of 10,000 cells per well into 24-well plates. Then, they were divided into three groups for exposure to different concentrations of HA as follows: control (no HA), 0.03 HA, and 0.3 HA groups (as mentioned above). Next, a combination of hyperosmolar solution (500 mOsM) and BAK (90 mM) was added to the medium to generate hyperosmolar conditions. Cells were monitored using a microscope with a camera attached to detect morphological changes, including decreased cell size, membrane blebbing, the formation of apoptotic bodies, and cell detachment, at 0 min, 5 min, and 15 min. Then, flow cytometry was conducted to examine the apoptosis rate, and real-time PCR was performed to measure the RNA expression levels of inflammatory cytokines, such as MMP-9 and TNF-*α*.

### 2.4. Animals

A total of 70 adult male Sprague-Dawley rats weighing approximately 250 g were purchased from the Animal Supply Centre of Sun Yat-sen University, Zhongshan School of Medicine. All procedures followed in this study were in accordance with the principles of the Association for Research in Vision and Ophthalmology (ARVO) Statement for the Use of Animals in Ophthalmic and Vision Research.

### 2.5. Corneal Epithelium-Injured Animal Models

The rats were anesthetized via intraperitoneal (i.p.) administration of kessodrate (10% chloral hydrate, 250 mg/kg) and were then placed beneath a stereoscopic microscope at 20x magnification. The right cornea of each rat was burned by a 3 mm^2^ round filter paper soaked with 100% heptanol; the filter paper was placed on the centre of the right cornea for 40 s, and then the cornea was rinsed with normal saline for 60 s. After injury, 70 rats were divided into 2 groups: a control group, in which the rats received normal saline eye drops (20 *μ*L) 4 times/d in the right eye, and a 0.3 HA group, in which the rats received 0.3% hyaluronate eye drops (20 *μ*L) 4 times/d in the right eye. The burned cornea of each animal was photographed on each day after injury to record disease progression and to analyse the healing rate. Real-time PCR and ELISA were performed to determine the expression levels of inflammatory cytokines (IL-1*β* and MMP-9) and repair factors (FN and CD44).

### 2.6. Real-Time PCR

Total RNA was isolated from individual corneas using TRIzol (Invitrogen, Carlsbad, CA) according to the manufacturer's recommendations, and it was quantitated using a NanoDrop 2000C spectrophotometer (Thermo Scientific, West Palm Beach, FL). One microgram of total RNA was reverse transcribed to produce cDNA, and the cDNA was amplified using SYBR Green Master Mix (Bio-Rad, Hercules, CA) according to the manufacturer's instructions. Primers for human MMP-9, human TNF-*α*, rat IL-1*β*, rat MMP-9, rat FN, and rat CD44 were purchased from SABiosciences (Frederick, MD), and the primer sequences are listed in Table 1. Quantitative real-time PCR was performed using a CFX96 real-time PCR system (Bio-Rad). Relative gene expression levels were calculated after normalization to the internal control *β*-actin.

### 2.7. Enzyme-Linked Immunosorbent Assay (ELISA)

Cytokine protein levels were selectively measured using ELISA kits (R&D Systems). For the* in vitro* experiments, HCEC supernatants were collected at 15 min. For the* in vivo* experiments, corneal samples were individually collected (*n* = 5/group/time) from the rats in the different groups at 1, 2, and 7 d after injury and homogenized in 0.5 mL PBS containing 0.1% Tween-20. All samples were collected and centrifuged at 13,000 rpm for 5 min, and the supernatants were collected. An aliquot of each supernatant was assayed in duplicate for measurements of the human FN and human CD44 levels, according to the manufacturer's instructions. The reported sensitivities of these assays are 0.6 ng/mL for human FN and 78.1 pg/mL for human CD44.

### 2.8. Flow Cytometry

Cell apoptosis was assessed by flow cytometry using an annexin V-fluorescein isothiocyanate apoptosis detection kit (BD) according to the manufacturer's instructions. Briefly, cells were pooled, washed, and resuspended in 500 *μ*L binding buffer, followed by the addition of 5 *μ*L annexin V-fluorescein isothiocyanate and 5 *μ*L PI. Then, the cells were incubated at room temperature away from light for 15 min and were subsequently analysed by flow cytometry (Beckman Coulter EPICS XL/MCL). Viable cells did not exhibit annexin V or PI staining, early apoptotic cells showed annexin V but not PI staining, and late apoptotic cells exhibited both annexin V and PI staining.

### 2.9. Statistical Analysis

Unpaired, two-tailed Student's *t*-test was used to determine the statistical significance of the ELISA and real-time PCR results and the corneal epithelium healing rates. The data were considered statistically significant at *p* < 0.05.

## 3. Results

### 3.1. Hyaluronate Promotes Cell Migration and Increases the Expression of Repair Factors

**Figure 1:**
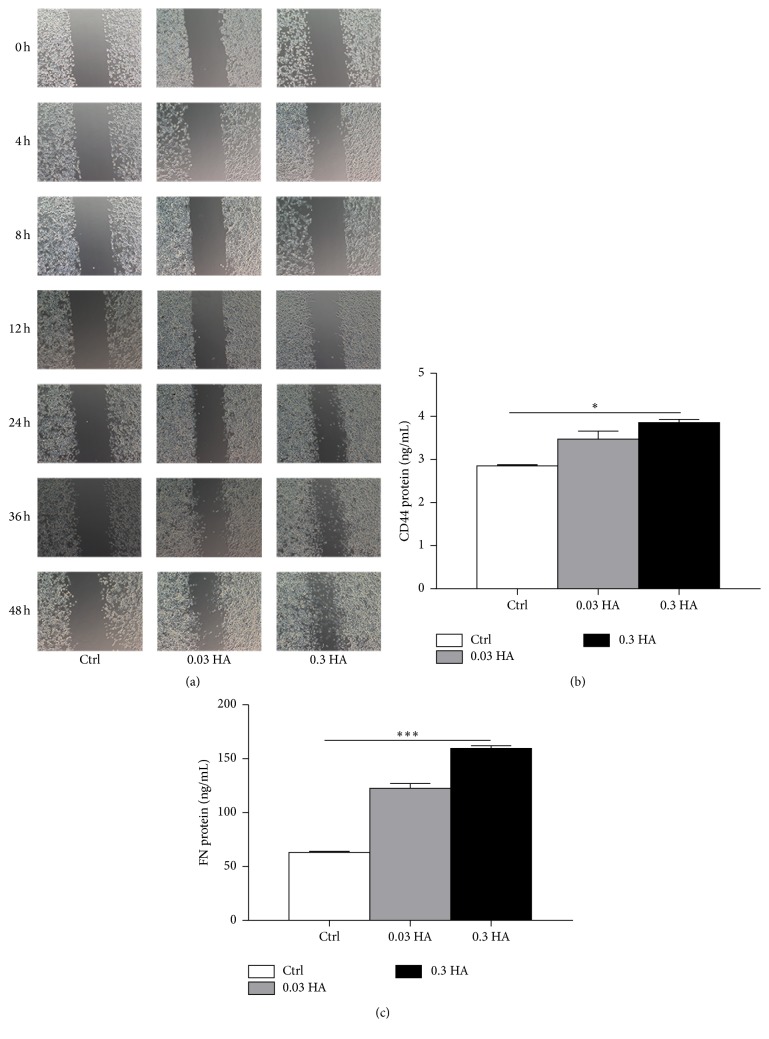
Hyaluronate promoted cell migration and increased the expression of repair factors. Microscopic images (a) of the repair of HCECs in the control, 0.03 HA, and 0.3 HA groups. CD44 (b) and FN (c) protein levels were examined in the control, 0.03 HA, and 0.3 HA groups. Magnification: ×20. The data are the mean ± SEM and represent individual experiments, each including 5 samples/group/time. ^*∗*^ *p* < 0.05; ^*∗∗∗*^ *p* < 0.001.

To determine the role of hyaluronate in cell migration, a culture insert was placed into culture dishes containing HCECs to form a gap. Then, different concentrations of hyaluronate were added to the culture medium, and the healing effects were observed using an inverted microscope. As shown in Figure 1(a), the gap was shortened gradually from 48 h after injury in the control group. In addition, the 0.03 HA group had a higher rate of cell proliferation, and an apparent new cell mass was observed at 36 h after injury, and the 0.3 HA group exhibited the highest proliferation rate, the formation of a new cell mass at 12 h after injury, and the highest rate of new cell formation at 48 h after injury.

Moreover, we determined the protein expression levels of Fibronectin (FN) and CD44. The mean FN protein expression level (Figure 1(b)) in the control group was 63.5 ng/mL, and it increased to 127 ng/mL in the 0.03 HA group and to 162 ng/mL in the 0.3 HA group, which was 2 times more than that in the control group (*p* < 0.05). The mean CD44 protein expression levels (Figure 1(c)) were 2.92, 3.77, and 3.92 ng/mL in the control, 0.03 HA, and 0.3 HA groups, respectively. The mean CD44 protein expression level was the highest in the 0.3 HA group (*p* < 0.001). The results shown in Figure 1 indicate that hyaluronate efficiently promoted wound closure and increased the expression of repair factors, especially in the 0.3 HA group.

### 3.2. Hyaluronate Decreases BAK-Induced Cell Apoptosis and the Expression of Inflammatory Cytokines

**Figure 2:**
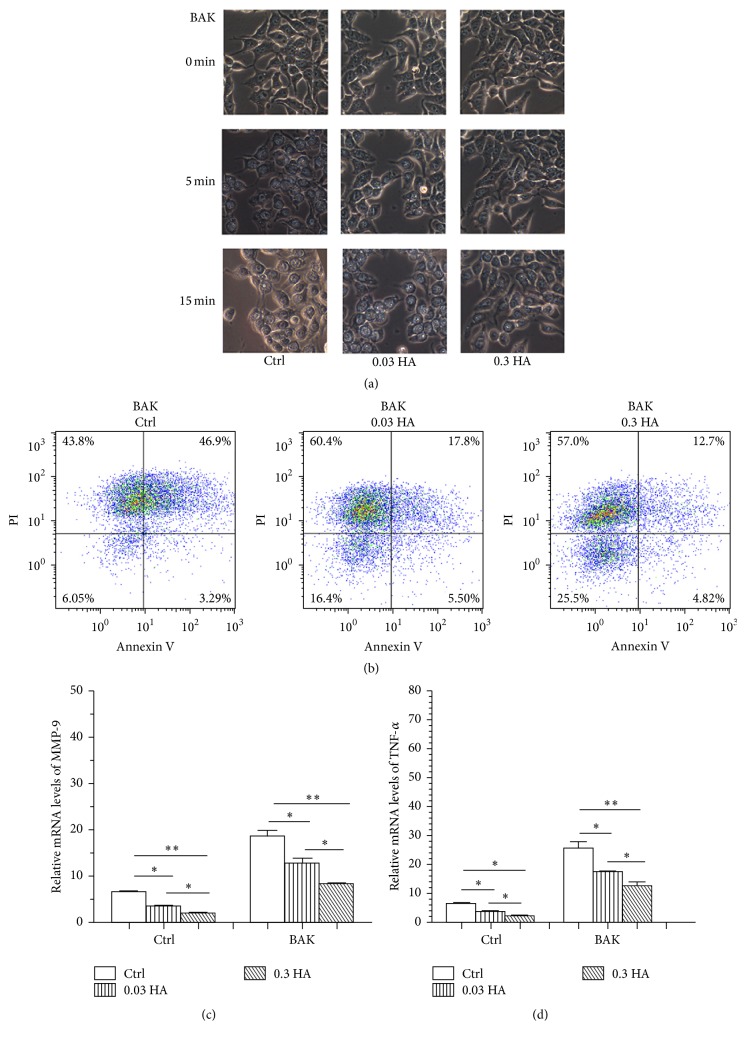
Hyaluronate decreased BAK-induced cell apoptosis and the expression of inflammatory cytokines. Microscopic images (a) of the changes in cell morphology in response to the hyperosmolar conditions were examined in the control, 0.03 HA, and 0.3 HA groups. Flow cytometry (b) was performed to analyse cell apoptosis, and real-time PCR (c) and (d) were used to compare the expression levels of MMP-9 and TNF-*α* among the three groups. Magnification: ×100. ^*∗*^ *p* < 0.05; ^*∗∗*^ *p* < 0.01; ^*∗∗∗*^ *p* < 0.001. The data are the mean ± SEM and represent individual experiments, each including 5 samples/group/time.

To explore the potential protective role of hyaluronate in BAK-induced cell apoptosis, NaCl and BAK were added to the medium to generate hyperosmolar conditions, and changes in cell morphology were observed using a microscope. As shown in Figure 2(a), cells in the control group exhibited clear decreases in cell size and cytoplasmic retraction after 5 min of hyperosmolar stimulation, those in the 0.03 HA group were inactivated and round in size at 15 min, and those in the 0.3 HA group were still relatively active at 15 min. Moreover, flow cytometry (Figure 2(b)) revealed that the 0.3 HA group had the lowest cell apoptosis rate (12.7% early apoptotic cells and 4.82% late apoptotic cells), followed by the 0.03 HA group (17.8% early apoptotic cells and 5.50% late apoptotic cells), and it revealed that the control group had the largest number of apoptotic cells (46.9% early apoptotic cells and 3.29% late apoptotic cells). Real-time PCR (Figures 2(c) and 2(d)) demonstrated that stimulation by BAK led to the upregulation of the expression of inflammatory cytokines, such as MMP-9 and TNF-*α*, compared with the control group. Hyaluronate downregulated their expression, and the 0.3 HA group showed the lowest inflammatory cytokine levels among the three groups. Taken together, these results revealed that hyaluronate facilitated reductions in cell apoptosis and inflammatory response induced by the hyperosmolar conditions.

### 3.3. Hyaluronate Accelerates Corneal Epithelial Wound Healing in a Heptanol-Burned Model

**Figure 3:**
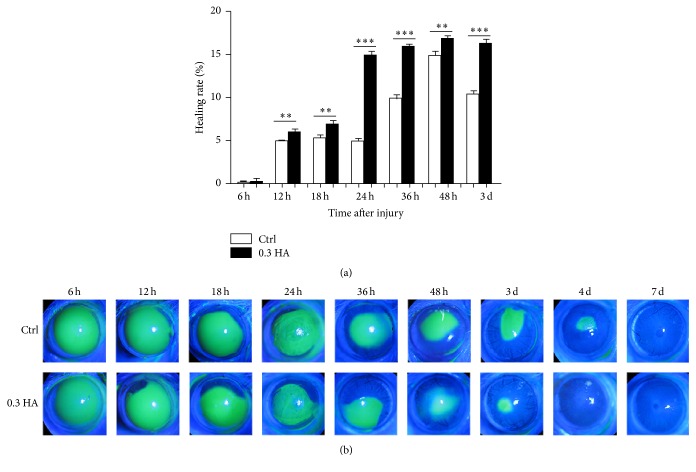
Hyaluronate accelerated corneal epithelial wound healing in the heptanol-burned model. SD rat corneal epithelial cells were removed by burning with heptanol. The healing rates (a) and fluorescein-stained corneal images (b) revealed the healing processes of the corneal lesions in the rats at 6 h, 12 h, 18 h, 24 h, 36 h, 48 h, 3 d, 4 d, and 7 d after injury in the control and 0.3 HA groups. Magnification: ×16, ^*∗*^ *p* < 0.05; ^*∗∗*^ *p* < 0.01; ^*∗∗∗*^ *p* < 0.001. The data are the mean ± SEM and represent individual experiments, each including 5 animals/group/time.

Based on the results of the* in vitro* study, we have demonstrated hyaluronate role in promoting corneal epithelial cell proliferation, and we have shown that higher concentrations of HA (0.3% HA) have enhanced effects. To further explore the role of hyaluronate* in vivo*, we generated an animal model with corneal epithelial defects in which rat corneas were burned with heptanol to damage corneal epithelial cells, and then 0.3% HA eye drops or normal saline was applied to the rats eyes 4 times/day to analyse the reparative effect of hyaluronate on corneal epithelial injury. The healing rates of the control group and the 0.3 HA group are compared in Figure 3(a). These rates were 4.958% in the control group and 6.04% in the 0.3 HA group at 12 h, with peaks of 14.9% in the control group and 17% in the 0.3 HA group at 48 h. The 0.3 HA group exhibited almost complete recovery at 4 d, while recovery was delayed to 7 d in the control group, indicating that the rate was significantly higher in the 0.3 HA group than that in the 0.3 HA group (*p* < 0.05). As shown in Figure 3(b), the rat corneal epithelial cells in the control group migrated gradually after injury and were intact at 7 d, while the 0.3 HA group exhibited almost complete corneal epithelial cell healing at 4 d, which demonstrated that 0.3% HA clearly accelerated the process of corneal epithelial repair. The results clearly demonstrate that hyaluronate promotes the healing of corneal epithelial cells* in vivo*.

### 3.4. Hyaluronate Modulates the Expression of Inflammatory Cytokines and Repair Factors* In Vivo*

**Figure 4:**
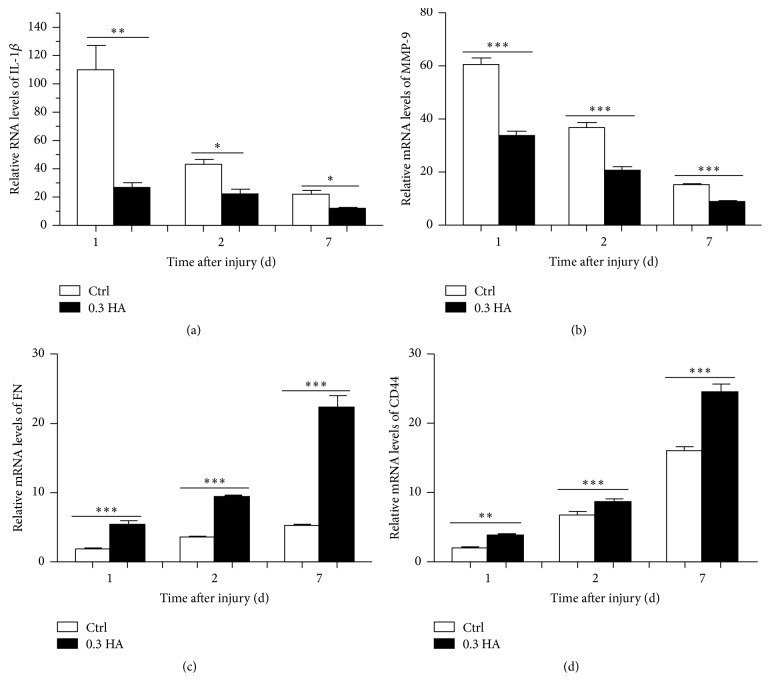
Hyaluronate decreased the expression of inflammatory cytokines and increased the expression of repair factors* in vivo*. The RNA levels of inflammatory cytokines, including IL-1*β* (a) and MMP-9 (b), and those of repair factors, including FN (c) and CD44 (d), were determined by real-time PCR at 1, 2, and 7 d after injury in the control and 0.3 HA groups. The data are the mean ± SEM and represent individual experiments, each including 5 animals/group/time. ^*∗*^ *p* < 0.05; ^*∗∗*^ *p* < 0.01; ^*∗∗∗*^ *p* < 0.001.

To explore the mechanism by which hyaluronate promotes the migration of corneal epithelial cells, we examined the expression of select inflammatory cytokines and repair factors by real-time PCR. At 1, 2, and 7 d after injury, the RNA expression levels of the inflammatory cytokines IL-1*β* and MMP-9 (Figures 4(a) and 4(b)) gradually decreased, and 0.3% HA clearly reduced these levels at all time points (all *p* < 0.05). For example, IL-1*β* expression was 5 times higher in the control group than in the 0.3 HA group at 1 d after injury, and MMP-9 expression was 2 times higher in the 0.3 HA group at 1 d after injury. Moreover, the repair factors FN and CD44 showed opposite trends as the inflammatory cytokines, exhibiting gradually increased expression after injury (Figures 4(c) and 4(d)). Administration of 0.3% HA significantly increased the expression of FN and CD44. For example, the expression of FN was 4 times higher and the expression of CD44 was 1.5 times higher in the 0.3 HA group than in the control group at 7 d after injury (all *p* < 0.05). These results suggest that 0.3% HA downregulates the expression of inflammatory cytokines and upregulates the expression of repair factors to attenuate inflammatory responses and improve corneal epithelial healing.

## 4. Discussion

A healthy corneal epithelium is essential for protecting the eye from infection and structural damage. An intact corneal epithelium is composed of five to seven layers of cells, and its healing process has been reported to include three separate phases [2, 18]. During the first phase, a provisional attachment complex, which is referred to as a focal contact, forms. The epithelial cells flatten and migrate as an intact sheet to cover the wound. During the second phase, cells distal to the original wound proliferate to repopulate the wound area, and cell stratification and differentiation occur. During the third phase, hemidesmosomes form, and extracellular matrix synthesis and reassembly occur [19, 20].

A large number of experimental studies on animals have confirmed that the healing process of corneal epithelial cells involves a complex series of interactions among extracellular matrix proteins, repair factors, and inflammatory cytokines [21–23]. Hyaluronate, an important substance involved in the healing process, has been reported to connect with various extracellular matrix proteins and to efficiently accelerate the healing process [24, 25]. Hyaluronate is found primarily in the extracellular matrix, and its biological functions include maintenance of liquid connective tissue, control of tissue hydration, and water transport. Moreover, the consistency and tissue-friendliness of hyaluronate allow it to serve as a viscosity-enhancing component in eye drops and as an adjuvant for eye tissue repair [26, 27]. However, it remains unclear whether exogenous hyaluronate promotes corneal epithelial cell migration and proliferation similar to endogenous hyaluronate. Additionally, the mechanism by which HA promotes migration and the dose-effect curve of HA remain unclear.

The process of proliferation is activated rapidly after injury in human corneal epithelial cells, and a large amount of hyaluronate is secreted from the cytoplasm for association with its receptors, such as CD44 and Fibronectin [16, 28]. CD44 is a transmembrane receptor for hyaluronate, and it is also capable of binding Fibronectin, laminin, and collagen I [29]. Fibronectin plays an important role in collagen deposition and stimulates the proliferation and differentiation of corneal epithelial cells and fibroblasts [30]. Evidence suggests that Fibronectin and CD44 play essential roles in providing a transient subepithelial matrix onto which migrating epithelial cells adhere during the frequent cycles of cleavage and attachment of these cells. These cells appear on wound surfaces within an hour after injury and have been found to contain cell receptor sites as well as binding sites for certain basement membrane components, including heparin sulphate and type IV collagen [16, 18, 31]. Once the process of corneal healing is initiated, the expression levels of hyaluronate and its receptors, Fibronectin and CD44, are increased.

Our study has determined the effects of different concentrations of hyaluronate on the migration and proliferation of HCECs* in vitro*, and we have concluded that 0.3% HA results in a higher proliferation rate than 0.03% HA. Therefore, we speculate that the levels of HA and its receptors are positively associated, achieving a “dose-efficiency” effect. Furthermore, the trends of increases in the CD44 and Fibronectin levels indicated that the higher concentration of hyaluronate allowed it to bind to its receptors to more efficiently accelerate the healing process. In addition, we used BAK and NaCl to induce hyperosmolarity and then examined cell changes that occurred under these conditions. BAK is known to be an inducer of oxidative stress and hyperosmolarity, and it can impair protective mechanisms and cause time- and dose-dependent increases in superoxide and ROS levels [32, 33]. We added different concentrations of hyaluronate to assess its protective role against BAK in human corneal epithelial cells. The results revealed that hyaluronate efficiently inhibited BAK-induced injury and delayed cell inactivation and that 0.3% HA exhibited better effects. We deduced that HA reduced the production and activity of proinflammatory mediators and matrix metalloproteinases and that it altered the behaviour of immune cells. These functions were manifested in the scavenging of reactive oxygen-derived free radicals, the inhibition of immune complex adherence to polymorphonuclear cells, the inhibition of leukocyte and macrophage migration and aggregation, and the regulation of fibroblast proliferation [33, 34]. Moreover, apoptosis is a process of programmed cell death that allows for the removal of abnormal cells [35]. Flow cytometry confirmed that hyaluronate efficiently decreased the percentages of late apoptosis and dead cells in agreement with the observation that HA alleviated the morphological changes of cells under adverse conditions.

To further verify the role of HA in promoting and repairing rat corneal epithelial cells, heptanol was used to remove the corneal epithelium to generate a corneal epithelium-injured animal model. The integrity of the corneal epithelial cells was dependent on cell-cell and cell-matrix interactions and on epithelial renewal, which has been widely demonstrated by* in vitro* studies. Considering the function of hyaluronate observed* in vitro* and its hygroscopic property [10], we deduced that it may also be important for modulating tissue hydration, osmotic balance, and cell renewal and differentiation* in vivo*. In our study, the complete repair of injured corneal epithelial cells took 7 days; for the control group, the healing rate was 15.39% at 24 h after injury, and it increased to 40.21% and 50.57% at 48 h and 72 h after injury, respectively. Comparatively, for the 0.3 HA group, the healing rate was 28.48% at 24 h after injury, and it increased to 61.52% and 77.88% at 48 h and 72 h after injury, respectively. Thus, the healing rate in the 0.3 HA group was clearly higher than that in the control group, demonstrating that hyaluronate promoted the repair of corneal epithelial cells.

Moreover, the inflammatory response was activated, and the expression of inflammatory cytokines, including IL-1*β* and MMP-9, was augmented. A possible relationship between inflammatory cytokines and ocular damage has been reported [36, 37]. In our study, the expression levels of inflammatory cytokines, such as IL-1*β* and MMP-9, gradually decreased following wounding, while those of the repair cytokines CD44 and FN were increased. It is possible that the proliferation and keratinization of epithelial cells and neovascularization are correlated with the activation of inflammatory cells, initiation of the MAPK signal transduction pathway, and increases in the secretion of inflammatory cytokines [38, 39]. Because hyaluronate promotes the healing of corneal epithelial cells and attenuates the inflammatory response, the protein and RNA levels of inflammatory cytokines, such as IL-1*β* and MMP-9, were lower in the 0.3 HA group, and those of repair factors, such as CD44 and Fibronectin, were higher compared with the control group.

To summarize, our study has demonstrated that hyaluronate accelerates the proliferation of human corneal epithelial cells and upregulates the expression of repair cytokines, thereby reducing apoptosis and downregulating the expression of inflammatory cytokines* in vitro*; in addition, it efficiently promotes the repair of ocular damage* in vivo*. Overall, this study provides a better understanding of and a promising therapeutic direction for hyaluronate in the treatment of ocular disease.

## Figures and Tables

**Table 1:** Nucleotide sequences of the specific primers used for PCR amplification.

Gene	Primer sequence (5′-3′)	
hMMP-9	AGGACAAAGCAGGATCACAGTT	F
	CCTGGGCAGATTCCAAACCT	R

hTNF-*α*	ATC AAT CGG CCC GAC TAT CTC	F
	GCA ATG ATC CCA AAG TAG ACC	R

h*β*-actin	GCT CCT CCT GAG CGC AAG	F
	CAT CTG CTG GAA GGT GGA CA	R

rIL-1*β*	CAT CTT TGA AGA AGA GCC CG	F
	GGG ATT TTG TCG TTG CTT GT	R

rMMP-9	CTTTGGGCTGCCCAACACACA	F
	GAAGCAGAATTTGCGGAGGTTTT	R

rFN	GAC CTG CAA GCC AAT AGC TGA GA	F
	TCG CCC AGA CAA GTA CAG TCC A	R

rCD44	GGA ATC AAG ACA GTG GAG TGA CCA CA	F
	GAC AGC AAT GCA GAC GGC AAG AAT	R

rGAPDH	AAT GCA TCC TGC ACC ACC AA	F
	TCA CGC CAC AGC TTT CCA GA	R
